# Overexpression of lncRNA-MEG3 inhibits endometrial cell proliferation and invasion via miR-21–5p/DNMT3B/Twist

**DOI:** 10.1016/j.clinsp.2023.100235

**Published:** 2023-06-29

**Authors:** Shaoyan Yang, Limei Feng, Qin Zhang, Lu Wu, Qinghua Zhao, Youfang Hou, Bo Yan, Suxian Zhang

**Affiliations:** a Department of Gynecology, The Second Affiliated Hospital of Kunming Medical University, Wuhua District, Kunming City, Yunnan Province, PR China; b Department of Pharmacy, The Second Affiliated Hospital of Kunming Medical University, Wuhua District, Kunming City, Yunnan Province, PR China

**Keywords:** lncRNA-MEG3, miR-21–5p, DNMT3B, TWIST, hESCs

## Abstract

Recent studies have found that lncRNA-MEG3(MEG3) plays an important role in the development of EMs (Endometriosis), but the specific mechanism needs to be further explored. This study aimed to investigate the effect of MEG3 on the proliferation, invasion of EMs cells. The authors used RT-qPCR to detect the expression of MEG3 and miR-21–5p in EMs tissues and hESCs cells, MTT and Transwell to detect cell proliferation and invasion, western blotting assay to detect the expression of DNMT3B and Twist, MSP to detect the methylation of Twist. The present study's detection results showed that MEG3 was lowly expressed in EMs tissues and hESCs cells, and overexpression of MEG3 could down-regulate miR-21–5p and inhibit endometrial cell proliferation and invasion. In addition, overexpression of MEG3 upregulated the expression of DNMT3B and promoted the methylation of TWIST. In conclusion, the present findings suggest that MEG3 is downregulated in EMs tissues, and overexpression of MEG3 can promote the activity of DNA methyltransferase DNMT3B by downregulating miR-21–5p, thereby promoting the methylation of Twist, downregulating Twist level to inhibits hESCs cells proliferation and invasion.

## Highlights

• MEG3 expressed exceptionally low in the ectopic endometrial.
• Overexpression of MEG3 inhibits hESCs proliferation and invasion.
• MEG3 is a ceRNA that has regulatory effects on miR-21–5p/DNMT3B axis.
• DNMT3B inhibits Twist expression after upregulation of MEG3 action.

## Introduction

Endometriosis (EMs), referred to as endometriosis, refers to a common gynecological disease in which the active endometrium grows outside the uterus and causes corresponding clinical symptoms [1,2]. EMs usually occur in women of childbearing age, and the incidence is increasing annually [3]. The ectopic endometrium usually involves the ovaries, uterosacral ligaments, pelvic organs, peritoneum, and vaginal rectal septum, which can cause dysmenorrhea, chronic pelvic pain, and abnormal menstruation, and in severe cases, it can lead to infertility [4]. The pathogenesis of EMs is extremely complex, involving many factors such as endocrine, inflammatory immunity, genetics, invasion and adhesion, angiogenesis, etc., but the exact etiology of EMs has still been unclear [5]. With the development of sequencing and omics, more and more results have shown that epigenetics (including DNA methylation, and histone modifications), non-coding RNA (ncRNA), and protein expression are different in ectopic endometrial tissue and normal endometrial tissue. and these differences may lead to changes in endometrial cell function [6]. Finding new methods for the diagnosis and treatment of endometriosis by studying the mechanisms of these differences is a current research hotspot.

Maternally Expressed Gene 3 (MEG3 for short) belongs to the LncRNA family [7]. It is widely expressed in normal tissues and cells as a tumor suppressor molecule. In some female ovarian cancer, [8] cervical cancer [9], endometrial cancer [10] and other tumor tissues and cells Expression is reduced or even absent. Previous studies have found that MEG3 inhibits the progression of endometrial cancer by inhibiting the phosphatidylinositol 3-kinase/mammalian rapamycin signaling pathway by binding to phosphatidylinositol 3-kinase. Although MEG3 has been widely and deeply studied in tumors, the role and related mechanisms of MEG3 in endometriosis remain unclear.

In this study, the authors found that lncRNA-MEG3 was lowly expressed in EMs tissues, and overexpression of lncRNA-MEG3 could inhibit hESCs proliferation and invasion. Further analysis showed that MEG3 inhibits the proliferation and invasion of hESCs by downregulating miR-21–5p and upregulating DMNT3B to promote Twist methylation.

## Materials and methods

### Clinical specimen collection

All samples were collected from the Department of Gynecology of The Second Affiliated Hospital of Kunming Medical University from July 2019 to January 2022. EMs patients (*n* = 24) with were confirmed by laparoscopy and histopathological examination. A total of 23 eutopic endometrium (EU) and 24 paired ectopic endometrium (EC) were collected from patients with EMs. 18 normal endometrium (NE) specimens were collected from healthy women who were hysteroscopically proven to be free of EM. All study women had normal menstrual cycles and had not received hormone therapy for the 3 months prior to surgery. Specimens for RNA and protein extraction were immediately stored in liquid nitrogen, the rest were used for histological examination. The study was conducted in accordance with the Declaration of Helsinki (as revised in 2013) and approved by the Ethics Committee of the Second Affiliated Hospital of Kunming Medical University (PJ-2021–110). All patients signed informed consent.

### Isolation and culture of primary HESCs

Human Endometrial Stromal Cells (HESCs) were isolated from the ectopic endometrium of women with endometriosis according to the method of Sung et al.[11]. Briefly, hESCs were isolated and maintained in monolayer cultures. HESCs were isolated and maintained in monolayer cultures. Minced endometrial tissue was washed, minced, and digested with collagenase and trypsin. After removing cellular debris, filter through a 38 µm sterile sieve to isolate stromal cells. Cells were plated in 6-well plates and assayed for cell purity by immunofluorescence.

HESCs were seeded on 6-well culture plates and cultured in DMEM/F12 medium containing 20% fetal bovine serum (Gibco, Carlsbad, USA), 100 μg/mL glutamine, 100 U/mL penicillin, and 100 μg/mL streptomycin (Sigma-Aldrich, St. Louis, MO, USA). The passage when cells reach 80% confluency.

### Cell viability

Cell viability was determined using the MTT assay (St. Louis, MO, USA). Briefly, hESCs were seeded into 24-well plates at a density of 2 × 10^4^ cells/mL for 24 h. 50 μL MTT solution (5 mg/mL prepared in phosphate buffered saline) was added to each well and incubated at 37 °C for 4 h. Next, removed the solution and added DMSO to the plates. The absorbance at 570 nm was measured using a plate reader.

### Western blotting

Total protein was extracted from tissue and hESC cells using RIPA Lysis Buffer (Invitrogen; USA), and the total protein concentrations were determined by using a BCA Protein Assay kit (Beyotime, China). Then, after boiling and denaturation, the extracted protein was separated by SDS-PAGE gel electrophoresis and transferred onto PVDF membranes. After that, the membranes were blocked with 5% Bovine Serum Albumin (BSA) for 1 h. After that, the membrane was incubated with primary antibodies (DNMT3B, Twist, and GAPDH [Abcam, USA]) at 4 °C overnight. After incubation, the membranes were washed three times with PBS and incubated with secondary antibodies at room temperature for 1 h. Then, the bands were visualized using an enhanced chemiluminescence kit (Bio-Rad Laboratories, Hercules, CA, USA).

### RT-qPCR

The total RNA of tissues and hESCs cells was isolated using the TRIzol reagent (Takara Kyoto, Japan), and the isolated total RNA was reverse transcribed using a cDNA Reverse Transcription Kit (Takara, Kyoto, Japan). Next, Real-time quantitative PCR amplifications were performed using SYBR® Premix Ex Taq™ (Takara, Kyoto, Japan). The relative abundance of MEG3 or miR-21–5p was determined by normalization to the level of GAPDH and U6, and analyzed using the 2^−ΔΔCt^ method. The primers were listed below: MEG3 forward: 5′-CTGCCCATCTACACCTCACG-3′, and reverse: 5′-ATCCTTTGCCATCCTGGTCC-3′; miR-21–5p forward: 5′-GCGCGTAGCTTATCAGACTGATG-3′, and reverse: 5′-CTGAAGTCGCCATGCAGATA-3′; U6 forward: 5′-ATGGACTATCATATGCTTACCGTA-3′, and reverse: 5′-GCTTCACGAATTTGCGTGTCATCCTTGC-3′. GAPDH forward: 5′-CAGGAGGCATTGCTGATGAT-3′, and reverse: 5′-GAAGGCTGGGGCTCATTT-3′.

### Cell invasion assays

Cell invasion measurements were conducted using a Transwell chamber coated with Matrigel (Corning, NY, USA). Treated hESC cells were cultured in the upper chamber of a Transwell plate for 12 h, and cells were washed 3 times with PBS. Next, 700 μL of medium containing 10% FBS was added to the lower chamber, and the Transwell plate was incubated in a 5% CO_2_ atmosphere at 37 °C for 48 h. After 48 h, the cells on the lower side were fixed in cold 3.8% formaldehyde and stained with 0.1% crystal violet. Finally, invaded cells were counted under the microscope (Olympus, Tokyo, Japan) and five randomly selected visual fields were randomly chosen to count.

### Methylation-specific PCR analysis

Genomic DNA was extracted from the tissues with the genomic DNA extraction kit (Qiagen, Dusseldorf, Germany). Next, the DNA was treated with sodium bisulfite. The methylated and unmethylated Twist were amplified using the following primers: Methylated: 5′-AAGGGCGAGAGAGTAGGTC-3′ (forward), 5′-TCCTAACCGCGATAACAAC-3′ (reverse); the unmethylated: 5′-AGAAGGGTGAGAGAGTAGGTT-3′ (forward), 5′-TCCTAACCACAATAACAACCC-3′ (reverse). The PCR-amplified products were separated in a 2% agarose gel and visualized.

### Immunohistochemical (IHC) staining

The paraffin sections were placed in a constant temperature oven at 65 °C, deparaffinized, hydrated, permeabilized, rinsed with 0.01 moL/L PBS (Tangier, China) solution, antigen retrieval, rinsed again with 0.01 moL/L PBS solution, blocked with 3% hydrogen peroxide, protect from light and incubate at room temperature for 30 min. Next, Sections were incubated with primary antibodies (Abcam, USA) for 1 h at room temperature. After washing with PBS 3 times, add a secondary antibody and incubated for 1 h. After washing with PBS 3 times, DAB was added to develop the color and observed under the microscope for 3‒10 min, the positive color was brown.

### Statistical analysis

Statistical analyses were performed by GraphPad Prism 7.0 software; *p* < 0.05 or *p* < 0.01 were considered statistically significant.

## Results

### The expression of MEG3, miR-21–5p and DNMT3B in endometriosis tissues

**Fig. 1:**
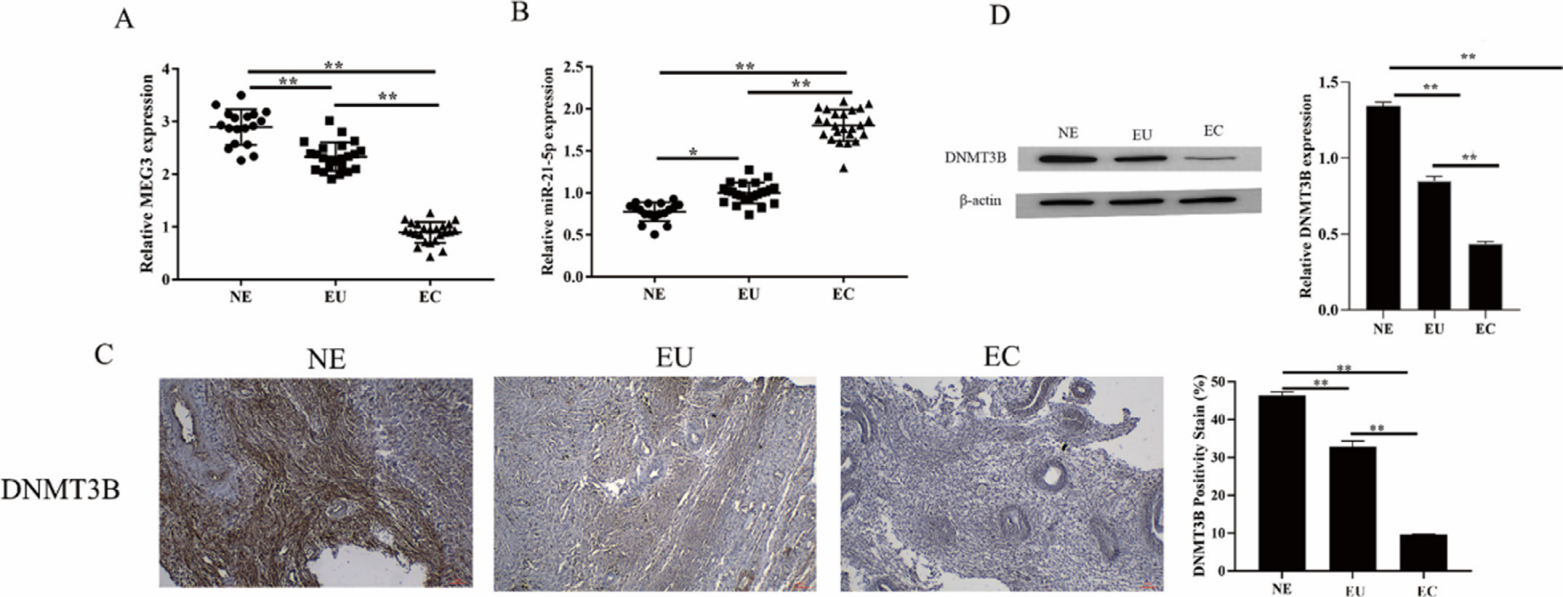
The expression of MEG3, miR-21–5p and DNMT3B in EMs tissues. The level of MEG3, miR-21–5p in tissues were measured by RT-qPCR (A‒B). The expression of DNMT3B was measured by IHC and western blotting (C‒D). Asterisks indicate statistical significance (*p < 0.05, **p < 0.01).

MEG3 has been confirmed to be involved in the occurrence of EMs[12]. To unfold the mechanism of MEG3 in EMs, the authors analyzed the expression of MEG3, miR-21–5p, and DNMT3B in clinical EMs tissues. The results showed that MEG3 and DNMT3B expressed exceptionally low in the ectopic endometrial group in EMs compared to the normal control group and the eutopic endometrial group in the EMs (Fig. 1A and Fig. 1C‒1D). In contrast, miR-21–5p exhibited the opposite findings, miR-21–5p expressed exceptionally high in the ectopic endometrial group in EMs compared to the normal control group and the eutopic endometrial group in the EMs (Fig. 1B). These results suggested that MEG3, miR-21–5p and DNMT3B might be involved in the development of endometriosis.

### Overexpression of MEG3 inhibits hESCs cell proliferation, invasion

**Fig. 2:**
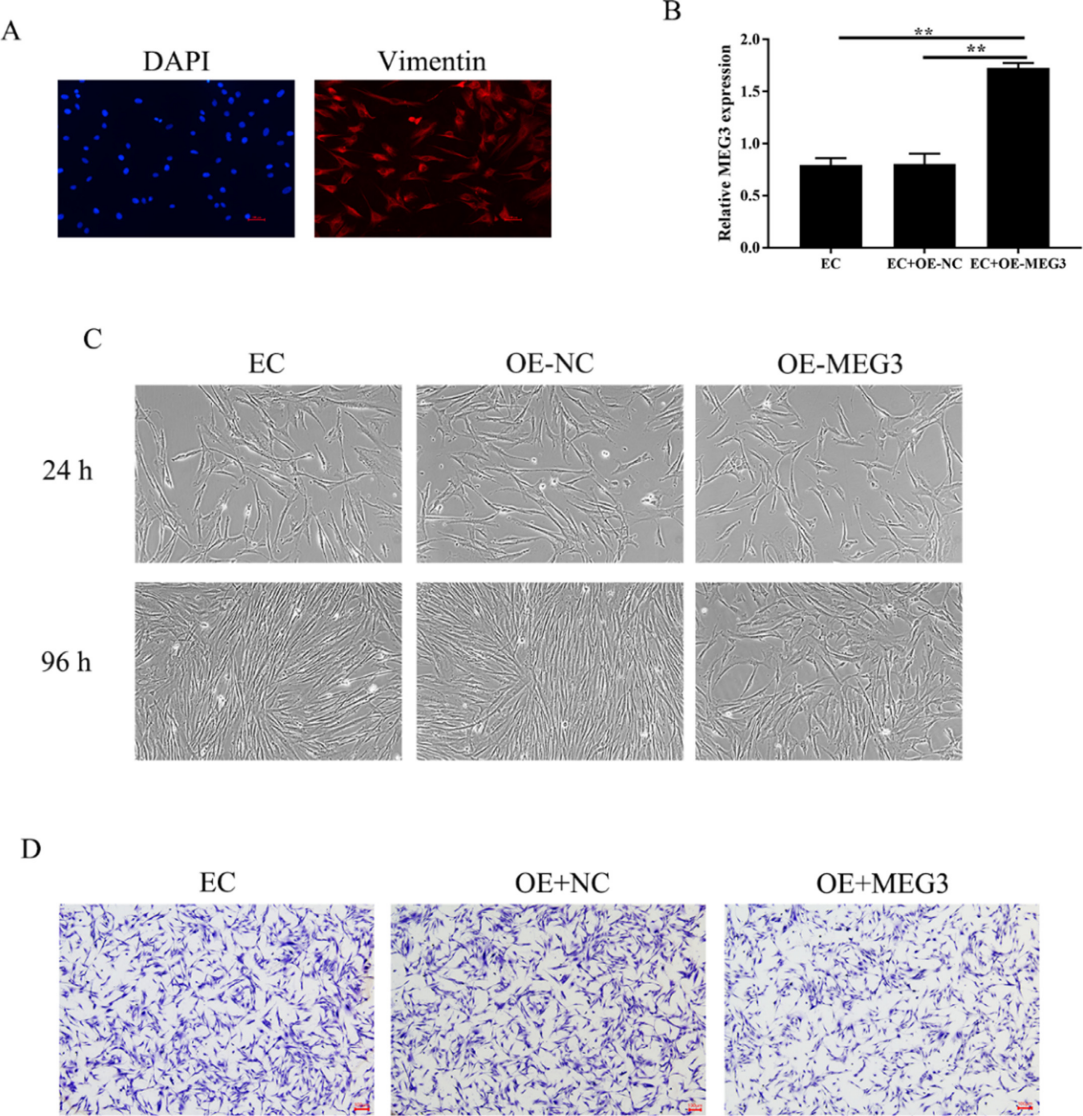
Overexpression of MEG3 inhibits hESCs cells proliferation, invasion. The expression of Vimentin in hESCs cells were measured by IF (the scale bar is 100 μm) (A). The level of MEG3 in hESCs cells were measured by RT-qPCR (B). The hESCs cells viability were measured by the MTT assay and microscope (200 ×) (C). The invasiveness of hESCs cells was evaluated by transwell invasion assay (the scale bar is 100 μm) (D). Asterisks indicate statistical significance (*p < 0.05, **p < 0.01).

Endometrial Stromal Cells (ESCs) continue to grow and spread in patients with EMs[13]. The authors isolated primary Endometrial Stromal Cells (hESCs) from ectopic endometrial and determine the success of separation by detecting the expression of vimentin (Fig. 2A). To clarify the effects of MEG3 on hESCs, the authors overexpression of MEG3 in hESCs and tested the efficiency of MEG3 overexpressing vector (Fig. 2B). Next, the authors examined the effect of MEG3 on cell viability and proliferation of hESCs. The cell growth of hESCs cells transfected with OE-MEG3 showed significantly lower compared with the OE-NC-transfected hESCs and untransfected hESCs (Fig. 2C). The effect of MEG3 on cell invasion was determined using transwell assays, transwell assays revealed that overexpression of MEG3 inhibited the invasion of hESCs cells in vitro As shown in Fig. 2D.

### MEG3 regulates the expression of miR-21–5p and DNMT3B

**Fig. 3:**
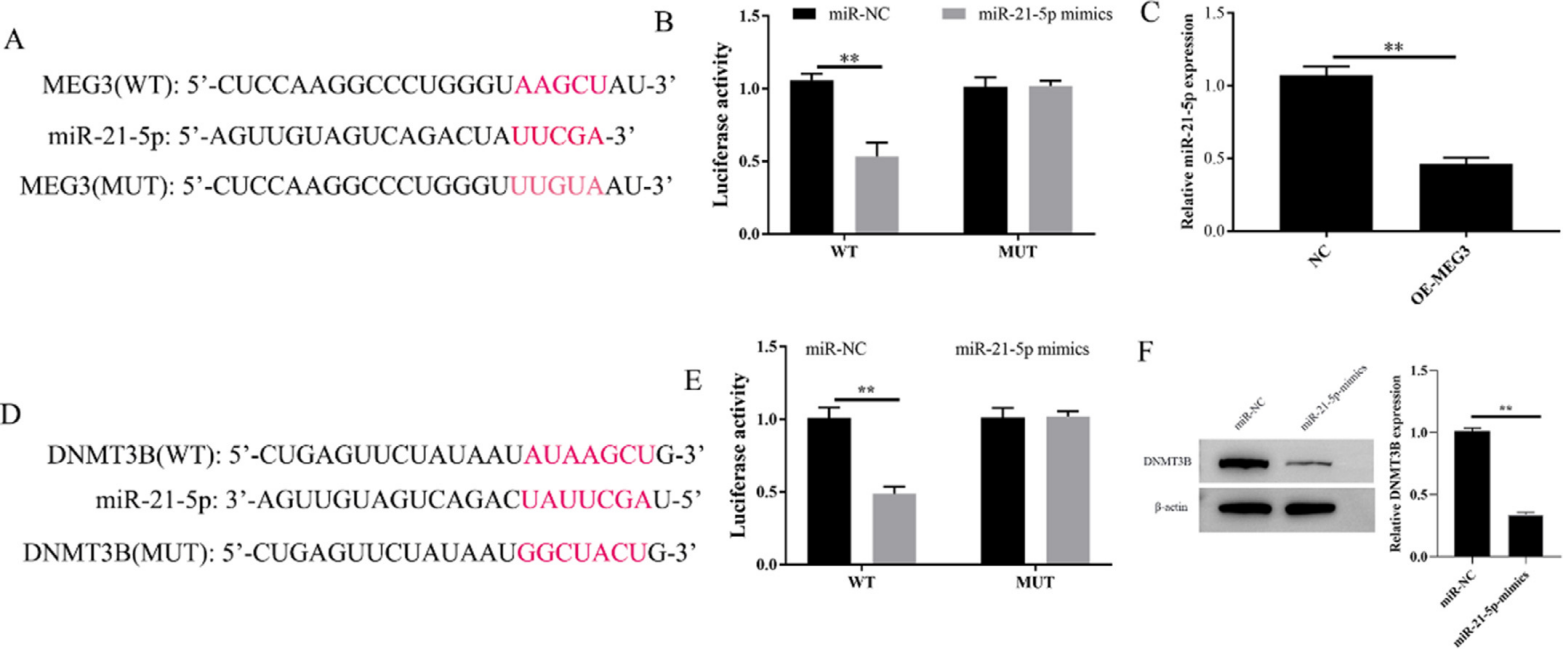
MEG3 regulates the expression of miR-21–5p and DNMT3B. The inferred binding regions between MEG3 and miR-21–5p, miR-21–5p and DNMT3B (A and D). Targeting relationship between MEG3 and miR-21–5p, miR-21–5p and DNMT3B determined by dual luciferase reporter genes (B and E). The level of miR-21–5p in cells were measured by RT-qPCR (C). The expression of DNMT3B was measured by western blotting (F). Asterisks indicate statistical significance (*p < 0.05, **p < 0.01).

To explore the potential downstream gene involved in the regulation of MEG3 on proliferation, and invasion in hESCs, the potential miRNA target genes of MEG3 were predicted using the miRCode databases. Bioinformatics showed that MEG3 has a sequence complementary to the region of miR-21–5p (Fig. 3A). The authors further performed a dual-luciferase reporter assay. The results showed that overexpression of miR-21–5p effectively inhibits the luciferase activity of Wild-Type (WT) MEG3 but not Mutant (MUT). In parallel, Overexpression of MEG3 downregulated the expression of miR-21–5p and also confirmed this interaction (Fig. 3B and C). On the other hand, through the Starbase database, the authors found that miR-21–5p and DNMT3B have a region-complementary sequence AAGCU, similar to MEG3 and miR-21–5p (Fig. 3D). Meanwhile, the dual-luciferase reporter assay and western blotting resulted further confirmed this interaction (Fig. 3E and F). Collectively, these finds revealed that MEG3 functions as a ceRNA to modulate the expression of miRAN-21–5p/DNMT3B.

### MEG3 inhibits hESCs cells proliferation, and invasion via miR-21–5p/DNMT3B

**Fig. 4:**
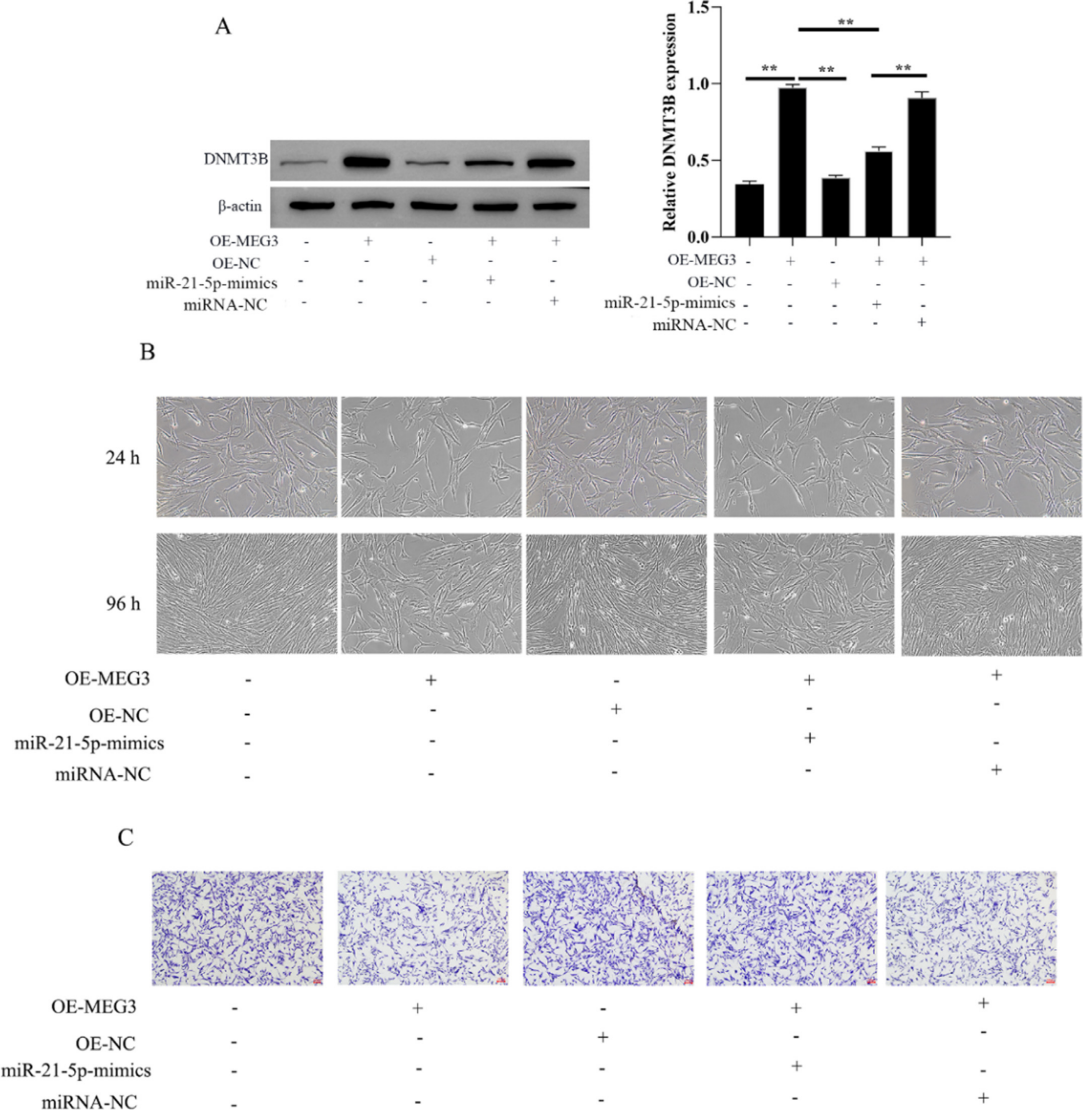
MEG3 inhibits hESCs cells proliferation, invasion via miR-21–5p/DNMT3B. The expression of DNMT3B was measured by western blotting (A). The hESCs cells viability were measured by the MTT assay and microscope (200 ×) (C). The invasiveness of hESCs cells was evaluated by transwell invasion assay (the scale bar is 100 μm) (D). Asterisks indicate statistical significance (*p < 0.05, **p < 0.01).

The authors evaluated the effect of MEG3 on the proliferation and invasion of hESCs cells via miR-21–5p/DNMT3B by transfecting miR-21–5p mimics in hESCs cells. The authors first investigated the expression of DNMT3B in OE-MEG3 and miR-21–5p mimics transfected and untransfected hESCs cells. The results showed that OE-MEG3 upregulated the expression of DNMT3B, in contrast, miR-21–5p mimics downregulated the expression of DNMT3B increased by OE-MEG3 (Fig. 4A). In addition, hESCs cells viability and proliferation showed that the miR-21–5p mimics downregulated the cells viability increased by OE-MEG3. Meanwhile, miR-21–5p mimics promoted cell invasion inhibited by OE-MEG3 (Fig. 4B and C). These finds revealed that MEG3/miR-21–5p/DNMT3B regulates hESCs cell proliferation and invasion.

### DNMT3B promotes twist methylation

**Fig. 5:**
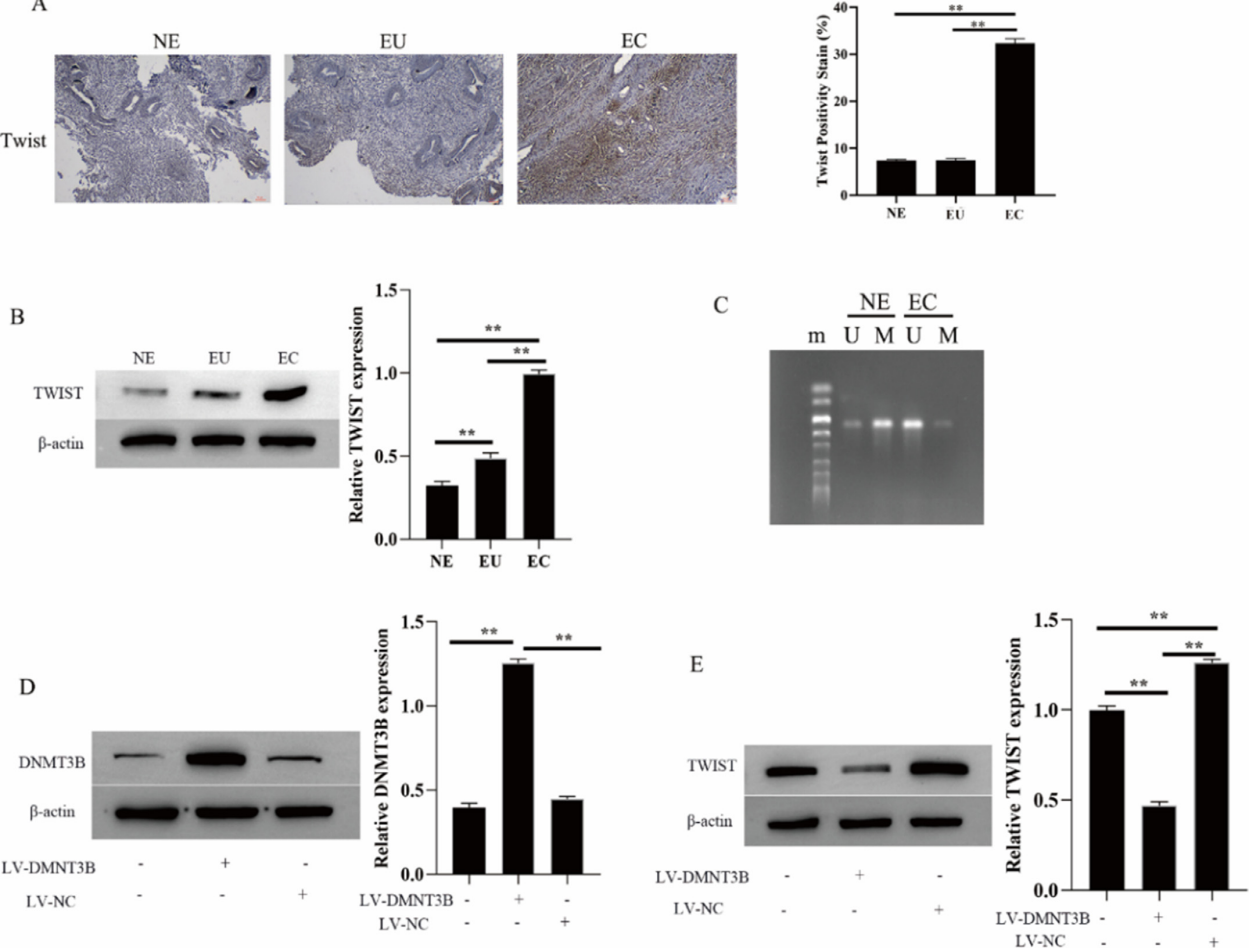
DNMT3B inhibits Twist methylation. The expression of DNMT3B was measured by IHC (A). The expression of DNMT3B and Twist were measured by western blotting (B, D and E). Methylation of Twist detected by MSP (C). Asterisks indicate statistical significance; m, Marker; U, Unmethylated, M, Methylated (*p < 0.05, **p < 0.01).

The twist is abnormally hypomethylated (highly expressed) in the development of EMs[14]. Thus, the authors further examined the methylation level of Twist in EMs and its relationship with the methyltransferase DNMT3B. IHC and Western blotting results showed that Twist expressed exceptionally high in the ectopic endometrial group in EMs compared to the normal control group and the eutopic endometrial group in the EMs (Fig. 5A and B). At the same time, Twist is hypomethylated in the ectopic endometrial group of EMs (U: Unmethylation, M: Methylation, Fig. 5C). Next, the authors investigated the regulation of Twist expression by DNMT3B by transfecting LV-DNMT3B in hESCs cells. The results showed DNMT3B expression is upregulated during LV-DNMT3B transfection, Meanwhile, the expression of Twist was downregulated (Fig. 5D and E). These results suggested that DNMT3B promotes Twist methylation and downregulates Twist expression

## Discussion

Although MEG3 has been confirmed to be related to EMs [12], the functional and specific mechanisms of MEG3 in EMs remain elusive. In the present study, it was verified that the expression of MEG3 is reduced in ectopic endometrial tissues. Simultaneously, overexpression of MEG3 inhibits hESC cell proliferation and invasion. In addition, the authors noticed that miR-21–5p and DNMT3B were also abnormally expressed in ectopic endometrial tissue. At the same time, miR-21–5p shared the same region complementary sequence AAGCU as MEG3 and DNMT3B. Further experiments found that overexpression of MEG3 inhibited the proliferation and invasion of hESCs by targeting the down-regulation of miR-21–5p and up-regulation of DMNT3B. On the other hand, DNMT3B promotes Twist methylation and downregulates Twist expression in hESCs cells. The present findings provide a molecular mechanism by which MEG3 inhibits the proliferation and invasion of hESCs by downregulating miR-21–5p and upregulating DMNT3B to promote Twist methylation.

In recent years, studies on EMs have found that endometrial cells in EMS patients have the ability to invade and proliferate similar to tumor cells[15,16]. In the research mechanisms of EMS, non-coding RNAs have been confirmed to play an important role in the invasion, migration, and proliferation of EMs cells[17,18]. Among them, lncRNAs and miRNAs are considered to have great potential in the early diagnosis and effective treatment of EMs[19,20]. Among the related lncRNAs, MEG3 was confirmed to be lowly expressed in female-related tumors, and as a tumor suppressor to inhibit the malignant progression of cancer cells in vitro and in vivo[21,22]. Simultaneously, MEG3 was reported to be down-regulated in ovarian endometriotic tissue[12], which is consistent with the present findings. In addition, the authors overexpressed MEG3 in hESCs isolated from ectopic endometrium and found that MEG3 inhibited the proliferation and invasion of hESCs, which also confirmed that MEG3 plays a key role in alleviating the progression of EMs. Previous studies have found that lncRNAs are involved in the development and regulation of EMs through the molecular axis acting by the endogenous competitive mechanism (Competing endogenous RNA, ceRNA). When examining the expression of miRNAs in EMs, the authors found that miR-21–5p was significantly upregulated in ectopic endometrial patient tissues, a result consistent with that of Park et al.[23]. More interestingly, MEG3 was shown to target and regulate the level of miR-21–5p[24]. At the same time, this study confirmed that miR-21–5p mimics down-regulated the cell viability increased by OE-MEG3, and promoted cell invasion inhibited by OE-MEG3.

DNA methylation, as one of the important epigenetic mechanisms of gene regulation, has been confirmed to be involved in the development of EMs[25]. DNA methylation regulates gene expression through DNA Methyltransferase 1 (DNMT1) and DNA Methyltransferase 3 (DNMT3A and DNMT3B)^26^, ^27^, ^28^. Here, the authors found that DNMT3B expressed exceptionally low in the ectopic endometrial group. This result is consistent with the results of previous studies[29]. In addition, notably, the authors found that the expression trend of MEG3 was consistent with that of DNMT3B, OE-MEG3 upregulated the expression of DNMT3B. This suggests that both DNMT3B and MEG3 play a protective role in the development of EMs, and at the same time, there may be a correlation between MEG3 and DNMT3B. Since the authors found that MEG3 regulates the expression of miR-21–5p in previous experiments, we detected the correlation between miR-21–5p and DNMT3B and found that DNMT3B is the target of miR-21–5p. Through cell proliferation and invasion assessments, the authors further confirmed that MEG3 inhibited cell proliferation and invasion by regulating miR-21–5p/DNMT3B.

The twist is a basic helix-loop-helix transcription factor[30]. Abnormal hypomethylation of Twist during protein methylation plays an important role in tumor metastasis by regulating EMT in epithelial cells[31,32]. Simultaneously, Twist is also considered to be one of the important mechanisms of the pathogenesis of EMs with malignant biological characteristics[33,34]. Some studies have found that the Twist gene promoter is hypomethylated in some regions of the ectopic endometrium by detecting three different tissues of ectopic endometrium, eutopic endometrium, and non-EMs eutopic endometrium. In parallel, the expression level of Twist was in the order of ectopic endometrium > eutopic endometrium > non-EMs endometrium[35]. Consistent with the present findings, the authors found that Twist was abnormally high in the ectopic endometrium group of EM. Meanwhile, Twist is hypomethylated in the ectopic endometrial group of EM. In addition, in the process of Twist protein methylation, the overexpression of methyltransferase (DNMT3B) is a prerequisite for causing DNA hypermethylation of various proteins including Twist[30,36]. In this study, the authors found that overexpression of DNMT3B in hESCs down-regulated the level of Twist. This also indicated that DNMT3B could affect the expression of Twist.

Taken together, the present findings provide evidence to show that MEG3 expressed exceptionally low in the ectopic endometrial, while overexpression of MEG3 inhibits hESCs proliferation and invasion. Furthermore, MEG3 is a ceRNA that has regulatory effects on the miR-21–5p/DNMT3B axis. DNMT3B inhibits Twist expression after upregulation of MEG3 action. In conclusion, targeting MEG3 could be a promising therapeutic strategy for EMs in the future.

## Data availability

All datasets presented in this study are included in the article. All data is real and guarantee the validity of experimental results.

## Authors’ contributions

Suxian Zhang conceived and designed this study. Shaoyan Yang, Limei Feng and Qin Zhang performed the cell experiments. Lu Wu, Qinghua Zhao, Youfang Hou, and Bo Yan collected and analyzed the data. Shaoyan Yang and Limei Feng arranged Figures. Suxian Zhang, Shaoyan Yang and Limei Feng drafted the initial manuscript. All authors have read and approved the final version.

## Conflicts of interest

The authors declare no conflicts of interest.
